# Research on Development Progress and Test Evaluation of Post-Quantum Cryptography

**DOI:** 10.3390/e27020212

**Published:** 2025-02-18

**Authors:** Meng Zhang, Jing Wang, Junsen Lai, Mingfu Dong, Zhenzhong Zhu, Ryan Ma, Jun Yang

**Affiliations:** 1 China Academy of Information and Communication Technology (CAICT), Beijing 100191, China; wangjing15@caict.ac.cn (J.W.); laijunsen@caict.ac.cn (J.L.); 2 KOAL Software Co., Ltd., Shanghai 200072, China; dmf@koal.com (M.D.); zhuzz@koal.com (Z.Z.); 3 VIAVI Solutions Inc., Beijing 100102, China; ryan.ma@viavisolutions.com (R.M.); jun.yang@viavisolutions.com (J.Y.)

**Keywords:** Post-Quantum Cryptography, development trends, testing and evaluation, standardization

## Abstract

With the rapid development of quantum computing technology, traditional cryptographic systems are facing unprecedented challenges. Post-Quantum Cryptography (PQC), as a new cryptographic technology that can resist attacks from quantum computers, has received widespread attention in recent years. This paper first analyzes the threat of quantum computing to existing cryptographic systems, then introduces in detail the main technical routes of PQC and its standardization process. Then, a testing and evaluation system for PQC is proposed and relevant tests are carried out. Finally, suggestions for future development are put forward.

## 1. Introduction

Quantum computing has been theoretically proven to have the ability to crack public key cryptography far beyond existing classical computing. In recent years, quantum computing technology has developed rapidly, especially with the introduction of quantum algorithms such as Shor’s algorithm [1] and Grover’s algorithm [2], which enable quantum computers to crack the integer factorization problem (IFP) and the elliptic curve discrete logarithm problem (ECDLP) in polynomial time. This directly threatens widely used public key encryption algorithms such as RSA and ECC.

With the advancement of quantum computing technology, the information security threat of public key cryptography cracking is becoming increasingly imminent. The potential capabilities of quantum computers will not only damage the existing digital trust system, but may also bring a retroactive “harvest now, decrypt later” threat to sensitive information that needs to be kept confidential for a long time. Therefore, responding to the information security threat of quantum computing has become a hot topic for global information security management agencies and industries.

In order to meet this challenge, researchers and cryptographers have begun to develop a new generation of cryptographic algorithms: Post-Quantum Cryptography (PQC). PQC provides a new type of public key cryptographic algorithm by upgrading the underlying mathematical difficulties. In August 2024, the National Institute of Standards and Technology (NIST) of the United States officially released the world’s first three PQC standards. The release of the PQC standard not only provides a clear evolutionary path for cryptographic regulators and industry users around the world to adopt quantum-safe encryption algorithms, but also promotes the further development of cryptographic technology. PQC research covers multiple technical routes such as lattice-based cryptography, encoding-based cryptography, and multivariate-based cryptography. These algorithms are believed to be theoretically able to resist attacks from quantum computers, but their practical applications still require further security assessment and standardization. At the same time, the transition from traditional encryption systems to PQC systems is an arduous and complex project, which requires advance planning, the formulation of upgrade migration timetables, and steady implementation.

In this paper, Section 2 discusses the information security risk challenges posed by the development of quantum computing. Section 3 elaborates on the key technical paths and standardization progress of PQC. In Section 4, the PQC evaluation system and method are constructed. On this basis, the relevant product prototypes are tested and evaluated. Finally, Section 5 puts forward suggestions for future development.

## 2. Information Security Risk Challenges Caused by Quantum Computing

Cryptographic algorithms mainly include symmetric algorithms, asymmetric algorithms, and hash algorithms. Symmetric cryptographic algorithms, also known as private key cryptographic algorithms, have the core feature that both communicating parties use the same key to encrypt and decrypt data. This algorithm is widely favored for its high efficiency, especially in scenarios such as data encryption transmission and database encryption storage. It can effectively ensure the confidentiality of data. Asymmetric cryptographic algorithms, or public key cryptographic algorithms, use a pair of different keys: the public key and the private key. The public key is used to encrypt information, while the private key is used for decryption. Asymmetric cryptographic algorithms’ encryption and decryption performance is slightly inferior to that of symmetric algorithms. Since private keys do not need to be shared, asymmetric encryption algorithms have richer application scenarios. The public key cryptography system is mainly based on the discrete logarithm problem (DLP), the large integer factorization problem (IFP) and the elliptic curve discrete logarithm problem (ECDLP). Among them, the RSA algorithm, which is based on IFP, and the elliptic curve cryptography (ECC) algorithm, which is based on ECDLP, are the most common in practical applications. However, the rapid development of quantum computing poses a serious threat to traditional public key cryptography systems such as RSA.

The security foundation of the RSA algorithm lies in the difficulty of IFP. The Shor algorithm, proposed by Peter Shor in 1994, can efficiently solve the IFP with polynomial time complexity on quantum computers, which directly threatens the security of the RSA algorithm. The core of the Shor algorithm is to use quantum Fourier transform to solve the period *r* of the function f(*x*) = *a^x^* mod *N*, and then find the prime factors *P* and *Q* of *N*. Although the physical realization of quantum computers cannot pose a substantial threat to large-scale RSA keys at present, with the advancement of quantum technology, this threat will gradually become a reality.

In addition to the Shor algorithm, converting the IFP into an optimization problem and using adiabatic quantum computing (AQC) [3] to crack the RSA public key is also a technical solution [4,5,6,7,8,9,10,11]. This solution can be implemented on quantum annealing (QA) machines and nuclear magnetic resonance (NMR) quantum computers based on adiabatic theory. At present, the integer 1,005,973 can be decomposed using quantum annealing machines [5]. Based on NMR quantum computing, integers of the order of hundreds can be decomposed [6]. Some schemes can even decompose integers of the order of tens of thousands [7] or hundreds of thousands [8]. Because these schemes use the special properties of prime factors, they are not universal.

Quantum computing also poses a security threat to the ECC algorithm, which is based on ECDLP. Studies have shown that the Shor algorithm can be used to attack the ECC encryption system [12]. Because the mathematical theory of the ECC algorithm is more complex than RSA and the Shor algorithm itself is designed for the problem of large integer decomposition, the attack scheme faces greater challenges in quantum circuit design and algorithm implementation. In addition, quantum computing is expected to accelerate the side channel attack (SCA) [13] against the ECC algorithm. On the hardware and software platforms where the encryption algorithm is deployed, some physical information will inevitably be leaked to the outside world during the working process. After the attacker obtains this information through various means, he can bypass the encryption algorithm and directly attack the encryption system. This method of attacking using leaked information is called a side channel attack. The side channel attack methods that can be used against the ECC algorithm are mainly divided into two types based on the quantum algorithm: differential power analysis using quantum annealing [14] and attack methods based on the Grover algorithm [15].

Compared with asymmetric encryption algorithms, symmetric encryption algorithms face smaller "quantum threats" [16]. For example, the application of the Grover algorithm [17,18,19] in an exhaustive attack can achieve quadratic acceleration and reduce the security strength of a symmetric key by half. It is believed that by simply doubling the key length of a traditional symmetric cryptographic algorithm, its security under a quantum computing model can be ensured to a certain extent.

**Figure 1:**
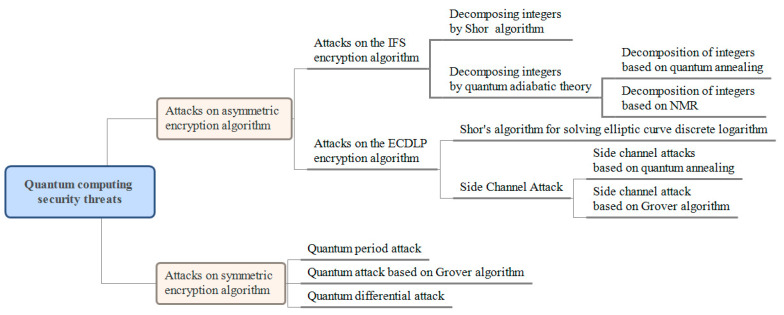
The security threat of quantum computing to traditional encryption system.

The security threat posed by quantum computing to a traditional encryption system is shown in Figure 1. In general, quantum computing has little impact on the traditional symmetric encryption system, while a traditional asymmetric encryption system is expected to be completely broken in theory and no longer capable of providing any security. Although quantum computing technology is not yet mature, once a breakthrough is made, it will seriously threaten the information security of many fields. Considering the long-term validity of sensitive data, quantum computing also has the “harvest now, decrypt later” threat.

## 3. PQC Development Status

In the field of cryptography, PQC is also called quantum-resistant cryptography (QRC). In a broad sense, quantum cryptography that uses the characteristics of quantum mechanics, such as quantum key distribution (QKD) [20], can also be considered a part of post-quantum cryptography because it has the ability to resist quantum attacks. In a narrow sense, post-quantum cryptography refers specifically to mathematical encryption technologies that can run on traditional computers and can resist future quantum computer attacks. PQC in this article refers to the narrow definition.

### 3.1. PQC Technology Research

According to the classification of underlying mathematical problems, there are currently five main technical routes for PQC algorithm research, namely lattice-based cryptography, encoding-based cryptography, multivariate-based cryptography, hash function-based cryptography, and curve homology-based cryptography. Different routes have their own characteristics and are suitable for different application scenarios.

#### 3.1.1. Lattice-Based Cryptography

Lattice-based cryptography is one of the most prominent and reliable PQC technologies. A lattice is a mathematical structure defined as a linear combination of integer coefficients of a set of linearly independent non-zero vectors (called lattice bases). The same lattice can have different lattice bases. Lattice cryptography is based on the difficulty of lattice problems such as the shortest vector problem (SVP) and the closest vector problem (CVP). The main features of lattice-based cryptography include:

- High security: lattice problems have extremely high complexity in high-dimensional space, and quantum computations are difficult to solve simply through parallel computing;
- Small key size: Compared with traditional public key cryptography, lattice-based cryptography has smaller public and private key sizes and faster calculation speeds;
- Wide application: lattice cryptography can be used to construct a variety of cryptographic primitives, such as encryption, digital signatures, and identity authentication;
- Representative algorithms: such as CRYSTALS-Kyber and CRYSTALS-Dilithium, which have been selected by NIST as PQC standards.

#### 3.1.2. Code-Based Cryptography

Code-based cryptography uses difficult problems in error-correcting code theory to build encryption algorithms. Coding theory is widely used for error correction in noisy channels. Code-based cryptography introduces a certain number of error codewords into the code, and correcting the error codewords or calculating the syndrome of the check matrix can be regarded as a difficult problem. Its main features include:

- Security foundation: The decoding difficulty is based on randomly generated linear codes, which provides security for cryptographic algorithms.
- Large key size: Compared with lattice-based cryptography, the public key size of code-based cryptography is larger, which may cause certain difficulties in practical applications.
- Fast encryption speed: Although the public key size is large, the encryption speed is fast.
- Representative algorithms: McEliece cryptography.

#### 3.1.3. Multivariable-Based Cryptography

Multivariable-based cryptography algorithms are based on the problem of solving high-order multivariable equations. Public key cryptography based on multivariate algorithms uses a set of quadratic polynomials on a finite field as a public key mapping. Its main security assumption is that solving a set of nonlinear equations on a finite field is an NP-hard problem. Its characteristics include:

- Fast signing speed: This type of algorithm is fast in signing and verifying signatures and consumes less resources.
- Large public key size: Although the signing speed is fast, the public key size is large.
- Applicable scenarios: Applicable to application scenarios in which public key transmission is not required frequently.
- Representative algorithms: Such as the HFEv-type GeMSS signature system and the UOV-type Rainbow signature algorithm.

#### 3.1.4. Hash Function-Based Cryptography

Hash function-based cryptography uses the anti-collision property of hash functions to construct encryption algorithms. When hash functions can resist strong collisions, digital signature algorithms based on hash functions can effectively resist attacks from quantum computing. Its main characteristics include:

- High theoretical security: The difficulty of hash functions is directly assumed to be equivalent to the complexity of ideal universal attacks.
- Large signature volume: The signature volume of this type of algorithm is usually large.
- Representative algorithms: XMSS and SPHINCS+, among which SPHINCS+ is the only hash-based encryption algorithm selected by NIST as one of its PQC standards.

#### 3.1.5. Curve Homology-Based Cryptography

Curve homology-based cryptography uses the homology relationship between elliptic curves to construct encryption algorithms; that is, for elliptic curves over finite fields, the homology (algebraic homomorphism) between given elliptic curves is calculated. Its characteristics include:

- Small public key and ciphertext size: Compared with other PQC algorithms, its public key and ciphertext size are very small.
- Low operating efficiency: The key generation, encryption, and decryption speeds are low, and it is not easy to implement on some devices with insufficient computing performance.
- Representative algorithms: the SIKE algorithm. Although it encountered attacks in the NIST evaluation, the homology problem itself was not cracked.

### 3.2. Current Status of PQC Standardization

NIST published a PQC overview in 2009. In 2012, NIST officially launched the PQC algorithm standard project. In 2016, NIST launched a global solicitation of PQC algorithms. In July 2022, NIST completed three rounds of elimination selection of PQC algorithms and officially announced four PQC algorithms that had directly entered the U.S. national standard-setting process. The performance characteristics are shown in Table 1.

On 13 August 2024, NIST officially released the world’s first three PQC standards, including:

- FIPS-203 (ML-KEM);
- FIPS-204 (ML-DSA);
- FIPS-205 (SLH-DSA).

These new standards can be divided into two major application areas: one is general encryption, which is used to protect information exchanged on public networks, and the other is protecting digital signatures, which are used for identity authentication.

In terms of general encryption, FIPS 203 is the main standard. This standard is based on the CRYSTALS-Kyber algorithm and has been renamed ML-KEM, which stands for Module-Lattice-Based Key Encapsulation Mechanism. The advantage of this standard is that it is a relatively small encryption key that can be easily exchanged and executed quickly.

In terms of digital signatures, FIPS 204 is the main standard. This standard uses the CRYSTALS-Dilithium algorithm, which has now been renamed ML-DSA, which stands for Module-Lattice-Based Digital Signature Algorithm. In addition, FIPS 205 is also a standard designed for digital signatures. This standard uses the Sphincs+ algorithm, which has now been renamed SLH-DSA, which stands for Stateless Hash-Based Digital Signature Algorithm. Since this standard uses a different mathematical method than ML-DSA, it can be used as a backup method if ML-DSA is found to have vulnerabilities. At the same time, NIST is continuing to evaluate the other two sets of algorithms, hoping that these algorithms can become backup standards to continuously ensure information security.

In addition to NIST, other standardization organizations in the United States and Europe, such as the Internet Engineering Task Force (IETF), the European Telecommunications Standards Institute (ETSI), the International Organization for Standardization (ISO)/International Electrotechnical Commission (IEC), the Institute of Electrical and Electronics Engineers (IEEE), etc., have also carried out PQC standardization research.

IETF has multiple working groups promoting the integration of PQC algorithms and traditional encryption protocols, as shown in Table 2. The focus is on the application of PQC technology in security protocols (such as TLS, IKE, etc.) as well as hybrid schemes of PQC and traditional encryption. For security and compatibility considerations, before fully migrating to the PQC algorithm, in the transition phase, the identity of a single entity is confirmed by using a post-quantum/traditional (PQC/T) or post-quantum/post-quantum (PQC/PQC) hybrid scheme, which is also applied to data protection. Based on the above considerations, IETF has given three methods for certificate migration: ① Multiple certificates, that is, the user has two certificates, namely a regular certificate and a PQC certificate. ② Hybrid non-composite certificate, that is, a regular public key and a PQC public key are provided on one certificate, and the CA completes both regular signatures and PQC signatures. This method can achieve backward compatibility, that is, after the full implementation of PQC migration in the future, the CA does not need to issue a new PQC certificate to the user. ③ Hybrid composite certificates, which encode multiple public and private key values into existing public and private key fields, using composite algorithms such as Dillithium-RSA. This method only requires adjustments to the cryptographic algorithm, avoiding modifications to the protocol.

ETSI has brought together the forces of industry and academia in Europe to carry out PQC migration-related work and form a cooperative ecosystem with NIST. In 2015, ETSI launched the global PQC algorithm flagship project PQCRYPTO and the PQC algorithm application project SAFCRYPT and invested a lot of money. By integrating the forces of multiple European universities and enterprises, ETSI has led and participated in the design of many algorithms.

The ISO/IEC working group follows the NIST trend, and NIST leads the relevant research work. The “Stateful Hash-Based Signature Mechanism” standard (ISO/IEC DIS 14888-4 [38]) is currently being formulated, covering the contents of standards such as IETF RFC8391 [39] (XMSS: Extended Merkle Signature Scheme) and RFC8554 [40] (Leighton-Micali Hash-Based Signatures). The standard is currently in the stage of soliciting comments. In addition, ISO/IEC plans to add content related to algorithms such as Kyber to the public key cryptography algorithm standard ISO/IEC 18033-2:2006 [41].

IEEE promoted the lattice-based NTRU algorithm as a standard in Std 1363.1 [42] as early as 2008. But due to the fact that the threat of quantum computing to traditional cryptographic technology had not yet become prominent at the time, it failed to attract enough attention. The subsequent NTRU algorithm entered the third round of NIST PQC algorithm selection but was eliminated at the end of the third round. In 2022, the IEEE launched P3172 “Recommended Practices for Post-Quantum Cryptography Migration” to discuss cryptographic agility, the implementation of hybrid mechanisms of traditional algorithms, and PQC algorithms.

## 4. PQC Testing and Evaluation

PQC technology evolution and application migration is a complex process involving multiple aspects such as technology, standards, and applications. Comprehensive testing and evaluation are required to ensure the smooth progress and security of algorithm research, product development, and upgrade migration.

### 4.1. PQC Testing and Evaluation System

**Figure 2:**
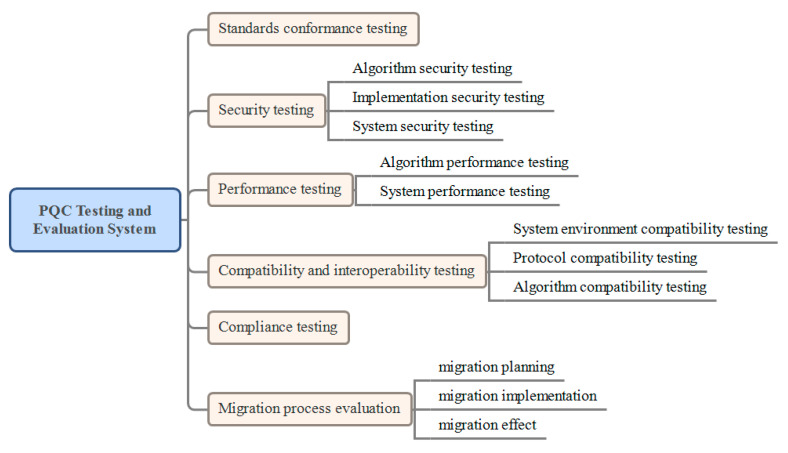
PQC Testing and Evaluation System.

Based on in-depth industry research, this article proposes a PQC testing and evaluation system for the first time, including standard compliance, security, performance, compatibility, compliance testing, and migration process evaluation, as shown in Figure 2.

Standards conformance testing: PQC-related algorithms, chips, product development, and upgrade and migration processes need to follow the relevant technical standards and specifications. Therefore, it is necessary to test whether the PQC algorithm and product comply with the PQC standards and specifications issued by the authority.

Security testing: It needs to be carried out at three levels: algorithm, implementation, and system. At the algorithm level, the PQC algorithm needs to be deeply analyzed for security, including an evaluation of the mathematical basis, encryption strength, and anti-attack capabilities of the algorithm. At the implementation level, the implementation of the PQC algorithm in actual applications needs to be tested for security, including a security review of the code and the detection of potential vulnerabilities and defects in the implementation process. At the system level, the migrated system needs to be fully tested for security, including the evaluation of the overall security architecture, security strategy, and security mechanism of the system to ensure that the overall security of the system is improved.

Performance testing: It needs to be carried out at two levels: algorithm and system. At the algorithm level, the encryption speed, decryption speed, key generation speed, and other performance indicators of the PQC algorithm need to be tested to evaluate its performance in different scenarios. At the system level, the performance of the migrated system needs to be fully tested, including the evaluation of performance indicators such as system throughput, response time, and resource consumption, to ensure that the system performance can still meet business needs after migration.

Compatibility testing: It is an important part of ensuring that encryption systems can operate and interact properly in different environments and platforms. Compatibility testing is particularly significant in scenarios in which multiple systems interact. Generally, it is carried out based on three aspects: system environment compatibility, protocol compatibility, and algorithm compatibility. System environment compatibility testing ensures that the PQC algorithm can run normally on different operating systems (such as Windows, Linux, macOS, etc.) and hardware platforms (such as x86 architecture PCs, ARM architecture mobile devices, high-performance servers, etc.). Protocol compatibility testing verifies whether the PQC algorithm can seamlessly integrate with existing encryption protocols (such as SSL/TLS, IPSec, etc.) and database systems (such as MySQL, Oracle, SQL Server, etc.). Algorithm compatibility testing verifies the compatibility between PQC algorithm and classic encryption algorithms (such as AES, RSA, etc.), as well as between different PQC algorithms, to ensure that they can work together in different application scenarios.

Compliance testing: PQC deployment, application, and upgrade migration also need to comply with the relevant laws and regulations. The testing needs to evaluate the regulatory compliance of the PQC algorithm and system to ensure that it complies with the provisions of laws and regulations in terms of data protection, privacy protection, and network security.

Migration process evaluation: It needs to be carried out at three levels: migration planning, migration implementation, and migration effect. At the migration planning level, PQC migration needs to formulate a detailed migration plan, including the goals, scope, steps, and timetable of the migration. The rationality and feasibility of the migration plan must be evaluated to ensure that the migration plan can be smoothly implemented. At the migration implementation level, during the implementation of a PQC migration, it is necessary to test and evaluate every aspect of the migration, including the progress, quality, cost, and other aspects of the migration, to ensure the smooth progress of the migration process. At the migration effect level, after the PQC migration is completed, it is necessary to conduct a comprehensive evaluation of the migration effect, including the evaluation of the post-migration system security, performance, compatibility, and other aspects to ensure that the migration has achieved the expected goals.

### 4.2. Testing Results

**Figure 3:**
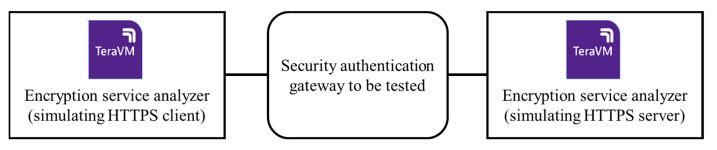
Test topology diagram.

We tested some key indicators of the security authentication gateway products that support PQC algorithms and verified the protocol conformance and system performance before and after the upgrade. In order to effectively test the protocol consistency and performance of the PQC gateway, we developed a PQC test tool based on VIAV’s protocol analyzer TeraVM. This tool supports not only traditional encryption algorithms, but also PQC algorithms, including Kyber512/768/1024, etc. It also supports IETF RFC 9370 [43], RFC 9242, RFC 8784, and other protocol standards. This tool supports emulations of both client encrypted flows and server traffic to be encrypted, as well as Per Tunnel, Per Flow KPI measurements. This tool helps evaluate the computational efficiency of PQC algorithms. This includes measuring factors such as encryption/decryption speed, key generation speed, and overall system performance. Ensuring that PQC algorithms are efficient is crucial for their practical adoption. The test topology is shown in Figure 3. We used VIAVI’s encryption service analyzer TeraVM to simulate an HTTPS client and HTTPS server to test the security authentication gateway device.

**Figure 4:**
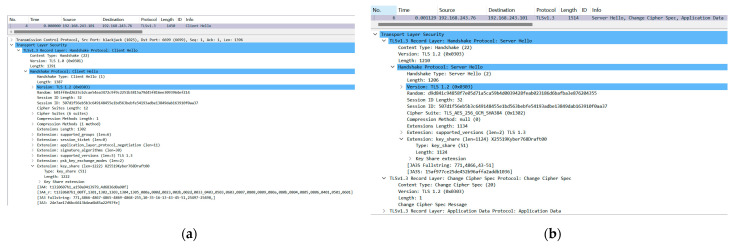
Results of TLSv1.3 handshake. (**a**) Client Hello protocol message. (**b**) Server Hello protocol message.

First, we verified the protocol conformance before and after upgrading the PQC function. Before the upgrade, the gateway device and analyzer were set to support the traditional encryption algorithms of X25519, prime256v1, and secp384r1. After the handshake between the client and server, the X25519 encryption algorithm was used. After upgrading to support the PQC algorithm, the gateway device and analyzer supported the traditional encryption algorithms of X25519, prime256v1, and secp384r1, as well as the PQC hybrid encryption algorithm of X25519Kyber768. After the handshake between the client and server, the X25519Kyber768 encryption algorithm was used, as shown in Figure 4.

**Figure 5:**
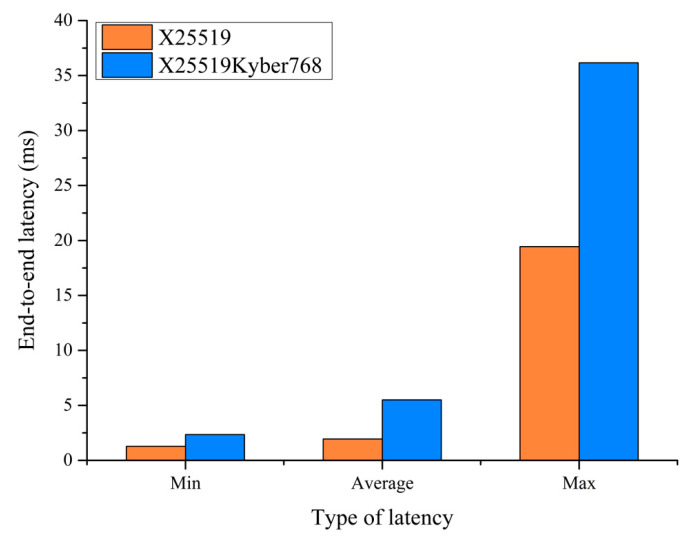
End-to-end latency results.

Next, we tested the transmission latency performance of the encryption service before and after the upgrade. The analyzer established TLS1.3 connection requests and simulated 40,000 users initiating HTTPS requests at a rate of no less than 2000 per second. The GET webpage size was set to 64 bytes. We recorded the end-to-end latency of the HTTPS services before and after the upgrade. The results are shown in Figure 5. It can be seen that the end-to-end latency of the PQC/traditional hybrid encryption scheme (X25519Kyber768) is significantly greater than that of the traditional encryption scheme (X25519). According to the IETF’s X25519Kyber768Draft00 hybrid post-quantum key agreement memo, for the client’s share, the Key_exchange value contains the concatenation of the client’s X25519 ephemeral share (32 bytes) and the client’s Kyber768Draft00 public key (1184 bytes). The resulting Key_exchange value is 1216 bytes in length. For the server’s share, the Key_exchange value contains the concatenation of the server’s X25519 ephemeral share (32 bytes) and the Kyber768Draft00 ciphertext (1088 bytes) returned from encapsulation for the client’s public key. The resulting Key_exchange value is 1120 bytes in length. The shared secret is calculated as the concatenation of the X25519 shared secret (32 bytes) and the Kyber768Draft00 shared secret (32 bytes). The resulting shared secret value is 64 bytes in length. Therefore, when executing the X25519Kyber768 hybrid encryption algorithm, the client and server conducted a dual round of key negotiation, and the key generation process for Kyber768 is comparatively sluggish compared with traditional encryption algorithms. This factor emerges as the principal contributor to the elevated end-to-end latency observed in PQC encryption services, surpassing that of traditional encryption methodologies.

Finally, we tested the long-term stability of the services when using PQC for encryption after the upgrade. In order to test the long-term stability of the gateway device under stressful working conditions, we specially designed the test services model. The test services model conforms to the dynamic characteristics of the traffic in the existing network as much as possible and creates pressure artificially. We used the analyzer to simulate multiple groups of users continuously and concurrently initiating HTTPS GET requests, and there are obvious differences in the size of GET pages for different users. On this basis, we designed a part of the group of users to continuously create and remove sessions. The analyzer configures multiple groups of users to continuously initiate HTTPS GET requests, where the GET webpage size includes 512 bytes, 100 kbytes, 500 kbytes, and 1 Mbyte. The specific configuration is as follows:

- 1000 terminals initiate GET 512-byte requests;
- 400 terminals initiate GET 100 K-byte requests;
- 200 terminals initiate GET 500 K-byte requests;
- 100 terminals initiate GET 1 Mbyte requests.

**Figure 6:**
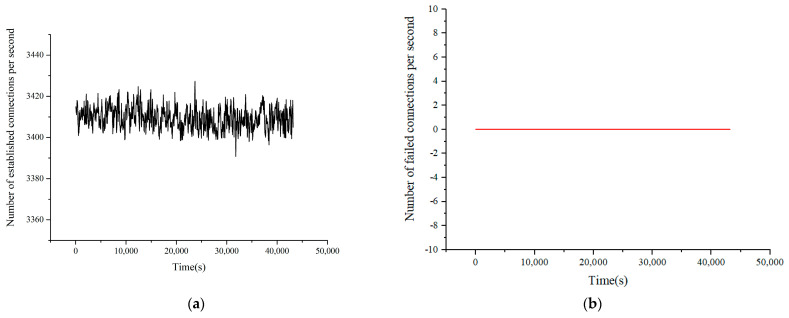
Results of connections per second. (**a**) Number of established connections per second. (**b**) Number of failed connections per second.

**Figure 7:**
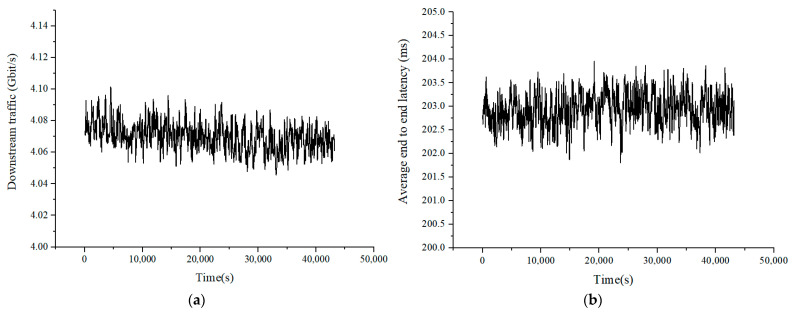
Results of traffic. (**a**) Results of downstream traffic. (**b**) Results of average latency.

Each TCP connection initiates a PQC-based encryption request. According to the above model, the number of new connections per second is set to 3000, the number of concurrent connections is set to 8000, and the throughput is guaranteed to be no less than 3 Gbps. All users come online and go offline continuously and generate traffic. The test was kept running for 12 h and the verification results were recorded. The results are shown in Figure 6 and Figure 7. The sampling period was 30 s. The results show that the device worked continuously for 12 h according to the verification conditions. The new connections, service throughput, and number of concurrent sessions remain stable, and no new connection failures occurred. The test results show that after upgrading the PQC function, the services’ transmission performance slightly decreased. However, the results of long-term testing under the stress testing model indicate that the upgraded PQC hybrid encryption algorithm can still ensure the stable transmission of service data. Multi-user dynamic establishment and deleting of sessions does not affect the normal transmission of other services. These results validate the feasibility of transitioning to PQC.

## 5. Discussion and Outlook

The evaluation system and methods proposed in this paper have the potential to be applied across various stages of PQC products, including design, development, production, deployment, and engineering acceptance. Furthermore, this paper has conducted experimental validation of PQC function upgrades for security authentication gateway products. Through rigorous testing, the protocol consistency, the end-to-end latency, and long-term stability of encryption service have been verified. The results indicate that hybrid algorithms combining PQC and traditional encryption can be effectively utilized in practical encryption systems, ensuring the stable transmission of encrypted services. This results not only substantiate the feasibility of PQC migration projects but also provide valuable guidance for the future deployment of PQC products.

PQC migration is an urgent and complex project that requires advance planning, careful design, and steady implementation. It is recommended to proceed in three steps:

1. Preparation: First, sort out the business system architecture, identify which cryptographic functions may be threatened by quantum computing, and form a migration list (including cryptographic technology characteristics, application scenarios, etc.). Evaluate the priority of migration based on the list content. Then, investigate the mainstream PQC algorithms and analyze their security, key size, latency, bandwidth, and applicable scenarios. Test their functions and performance to evaluate the impact of the algorithm on the system. For cryptographic functions that may be threatened by quantum computing, study the security compensation plan after the algorithm is replaced to ensure that the security is not reduced.
2. Product transformation: After the PQC algorithm standard is released, promote migration according to the priority of the migration list. First, under the leadership of industry regulators, pilot verification in key areas. Then gradually promote comprehensive migration and replacement, then finally achieve full product and full system support. Specifically, this includes: upgrading the digital certificate infrastructure of CA issuing agencies to support PQC algorithms; cooperating with units using certificates; and upgrading certificate issuance products, such as electronic signatures and certificate issuance management. After the certificate issuance products are compatible, upgrade the certificate application products, including software products such as encryption and decryption components and hardware products such as gateways and signature verification servers. Finally, upgrade the certificateless public key cryptographic products to ensure that all public key cryptographic algorithm products support PQC algorithms.
3. Industry promotion: The industry can first conduct pilot verification typical scenarios, collect feedback, and improve cryptographic products. After fully verifying the feasibility, PQC can be fully promoted and applied.

## Figures and Tables

**Table 1:** Performance characteristics of the PQC algorithm selected by NIST.

Type	The Name of the Algorithm	Math Problem	Performance Characteristics
Encrypt	Kyber	Lattice-based	With strong security and excellent performance, NIST predicts that this algorithm will be the first choice for most scenarios
Signature	Dilithium	Lattice-based	With strong security and excellent performance, NIST predicts that this algorithm will be the first choice for most scenarios
	Falcon	Lattice-based	Compared to Dilithium, the signature size is small (666 bytes for Falcon and 2420 bytes for Dilithium at 128 bits security strength) and the implementation complexity is higher (more gate count or memory may be required)
	Sphincs+	Based on hash	Compared with lattice-based algorithms, signatures are large in size and slow. It does not depend on the difficult problem of the lattice, and is a complementary choice

**Table 2:** PQC-related standard projects published or under development by IETF.

Working Groups	RFC/Draft	Main Content
LAMPS (Limited Additional Mechanisms for PKIX and SMIME)	RFC 8696 [21]	Using Pre-Shared Keys (PSKs) in CMS Digital Signatures
	RFC 8708 [22]	Hash-based signature algorithms based on Hierarchical Signature System (HSS) and Leighton-Micali signature (LMS) are used in CMS digital signatures
	draft-ietf-lamps-cms-kyber-07 [23]	Using Module-Lattice-Based Key Encapsulation Mechanism (ML-KEM) in CMS Digital Signatures (corresponding to [FIPS203])
	draft-ietf-lamps-kyber-certificates-06 [24]	Internet X.509 Public Key Infrastructure—Algorithmic identifier for Module Lattice-based Key Encapsulation Mechanism (ML-KEM) (corresponding to [FIPS203])
	draft-ietf-lamps-cms-sphincs-plus-17 [25]	Using the SLH-DSA Signature Algorithm in CMS Digital Signatures (Corresponding to [FIPS205])
	draft-ietf-lamps-dilithium-certificates-05 [26]	Internet X.509 Public Key Infrastructure: Module-based Digital Signature Algorithm (ML-DSA)-based Algorithm Identifier (corresponding to [FIPS 204])
TLS (Transport Layer Security)	RFC 8773 [27]	TLS 1.3 extension for certificate-based authentication using an external pre-shared key
	draft-ietf-tls-8773bis-03 [28]	The TLS 1.3 extension is used to use certificates with external pre-shared keys
	draft-ietf-tls-hybrid-design-11 [29]	Use post-quantum/traditional hybrid key exchange in TLS 1.3
IPSECME (IP Security Maintenance and Extensions)	RFC 8784 [30]	Mix pre-shared keys in Internet Key Exchange Protocol version 2 (IKEv2) for post-quantum security
	RFC 9242 [31]	Intermediate exchange in Internet Key Exchange Protocol version 2 (IKEv2)
COSE (CBOR Object Signing and Encryption)	draft-ietf-cose-dilithium-05 [32]	Tig-based Digital Signature Standard (ML-DSA) (FIPS 204)-based serialization of JSON Object Signing and Encryption (JOSE) and CBOR Object Signing and Encryption (COSE)
	draft-ietf-cose-sphincs-plus-05 [33]	SLH-DSA (FIPS 205)-based JSON Object Signing and Encryption (JOSE) and CBOR Object Signing and Encryption (COSE) serialization
PQUIP (Post-Quantum Use In Protocols)	draft-ietf-pquip-hybrid-signature-spectrums-05 [34]	Design and security objectives of different hybrid signature schemes
	draft-ietf-pquip-pqc-engineers-06 [35]	The impact of cryptography-related quantum computers (CRQCs) on existing systems and the challenges involved in the transition are presented
	draft-ietf-pquip-pqt-hybrid-terminology-05 [36]	Terminology for post-quantum/traditional hybrid schemes
	draft-wiggers-hbs-state-01 [37]	Hash-based signatures: state and backup management

## Data Availability

Data is contained within the article.
